# Nanostructured fuzz growth on tungsten under low-energy and high-flux He irradiation

**DOI:** 10.1038/srep10959

**Published:** 2015-06-16

**Authors:** Qi Yang, Yu-Wei You, Lu Liu, Hongyu Fan, Weiyuan Ni, Dongping Liu, C. S. Liu, Günther Benstetter, Younian Wang

**Affiliations:** 1 School of Physics and Materials Engineering, Dalian Nationalities University, Dalian 116600, People’s Republic of China; 2 Key Laboratory of Materials Physics, Institute of Solid State Physics, Chinese Academy of Sciences, P. O. Box 1129, Hefei 230031, P. R. China; 3 Fujian Key Laboratory for Plasma and Magnetic Resonance, Department of Electronic Science, Aeronautics, School of Physics and Mechanical & Electrical Engineering, Xiamen University, Xiamen, 361005, People’s Republic of China; 4 Faculty of Electrical Engineering and Media Technology, Deggendorf Institute of Technology, Deggendorf 94469, Germany; 5 School of Physics and Optoelectronic Technology, Dalian University of Technology, Dalian 116024, People’s Republic of China

## Abstract

We report the formation of wave-like structures and nanostructured fuzzes in the polycrystalline tungsten (W) irradiated with high-flux and low-energy helium (He) ions. From conductive atomic force microscope measurements, we have simultaneously obtained the surface topography and current emission images of the irradiated W materials. Our measurements show that He-enriched and nanostructured strips are formed in W crystal grains when they are exposed to low-energy and high-flux He ions at a temperature of 1400 K. The experimental measurements are confirmed by theoretical calculations, where He atoms in W crystal grains are found to cluster in a close-packed arrangement between {101} planes and form He-enriched strips. The formations of wave-like structures and nanostructured fuzzes on the W surface can be attributed to the surface sputtering and swelling of He-enriched strips, respectively.

Tungsten (W) as one of the best plasma-facing materials for ITER will suffer from bombardments of large flux (10^22^−10^24^/m^2^·s) and low-energy (tens of eV to hundreds of eV) ions of helium (He) and hydrogen ions^1^,^2^. The bombardments of W by the high-flux and low-energy He ions often lead to serious irradiation damages, such as the formation of voids, wave-like microstructures, or nanostructured fuzzes (nano-fuzzes) at the surface^3^,^4^,^5^,^6^. The growth of nano-fuzzes in W is a major concern because it may cause serious etching of W surface and shorten the lifetime of the W components in ITER. The enhanced erosion and dust formation of W nano-fuzzes can have fatal influence on the stability of fusion plasmas in the fusion reactor^7^.

Recently, the formation mechanism of voids and nano-fuzzes has become a subject of intensive investigations, and the surface changes of W have been thought to result from the near-surface He trapping at defect sites^4^. Kajita *et al.*^7^ proposed that nanometer-sized He bubbles formed in the W surface layer can grow and coalesce at an elevated temperature. These small He bubbles can serve as seeds for the formation of voids and nano-fuzzes. The fuzz-formation mechanism was proposed to consist of the following three elementary processes^7^,^8^: (1) penetration of incident He atoms into W, (2) diffusion of He atoms and their trapping at thermal vacancies, and (3) formation and aggregation of He bubbles. Iwakiri *et al.*^6^ proposed that He-vacancy complexes should be formed by continuous absorption of He and ejection of W self-interstitials. Impurity atoms were proposed to act as trapping centers for He atoms, which formed bubbles by ejecting W atoms from their lattice sites. Nishijima *et al.*^9^ proposed that He atoms diffusing inside W can be easily trapped in vacancies and become nanometer-sized bubbles. An increase in the internal pressure of these trapping sites results in the mutation of crystal lattices. Yoshida *et al.* proposed that the nucleation of interstitial loops in the stress field of a He-vacancy complex can greatly contribute to the formation of He bubbles^10^, and finally results in the nanostructured surfaces of W. Although the formation processes of voids, wave-like microstructures, and nano-fuzzes have been widely discussed in early studies, their evolution mechanism is still not fully understood.

Here, we report a comparative study of nanostructured surfaces and near-surface defect distributions of crystalline W exposed to low-energy and high-flux He ions. This study reveals that the formations of wave-like microstructures and nano-fuzzes of He^+^-irradiated W crystal grains can be attributed to the surface sputtering and swelling of He-enriched W crystal faces. Our calculations confirm the existence of the stable He monolayers between W {101} planes.

Polycrystalline W specimens with a purity of 99.99% (Honglu Corporation, China) were cut into pieces with a dimension of 10 × 10 × 2 mm^3^. The polishing machine (UNIPOL-1200M) was used for the polishing experiments. W surfaces were mechanically mirror-polished to a surface roughness of <0.1 μm. After polishing, these W specimens were annealed at 1373 K for 2 h in vacuum with a background pressure of 10^−5^ Pa to relieve internal stress and reduce the large concentration of defects. The large-power RF plasma system newly built in our lab was utilized to irradiate these W specimens. By this system, large-power (P_max_ = 10 kW) RF plasmas were generated inside the quartz tube (10 cm in diameter, 18 cm in length) by inductively coupling of 2 MHz RF to the water-cooled RF coils. During He^+^ irradiation, RF power and He pressure in the quartz tube were fixed at 8.0 kW and 30 Pa, respectively. The electron density and temperature in the plasma source were 5 × 10^18^ m^−3^ and 4.5 eV, respectively, and the He^+^ flux remained constant at 2.5 × 10^22^ ions/m^2^·s. The He^+^ fluence varied from 1.0 × 10^25^ to 1.0 × 10^27 ^ions/m^2^. The W specimen placed on a specially designed specimen holder was inserted into the high-density RF plasma. The bias voltage of −50 V was applied to the W specimen, resulting in an incident energy of 70 eV/He^+^ when taking account the plasma potential of 20 V measured by a Langmuir probe. Specimen temperature was measured with an infrared STL-150B pyrometer. The infrared pyrometer was fixed outside the plasma source in the ambient air. The infrared pyrometer collected a portion of the thermal radiation emitted by the W specimen. During He^+^ irradiation, the W surface temperature remained constant at 1400 K.

After He^+^ irradiation, SEM (Hitachi S-4800) was utilized to observe the surface microstructures of W specimens. Scanning electron microscopy-electron backscatter diffraction (SEM-EBSD) measurements were performed using Nordlys Max2. Beam conditions were 20 keV, 5.0 nA, with a 13 mm working distance. Grain calculations were based upon the definition that a >15° pixel-to-pixel misorientation defines a grain boundary.

Conducive atomic force microscope (CAFM) measurements have been performed to reveal the microstructures of irradiated W specimens. Previously, CAFM (Veeco DI 3100) has been utilized to detect the nanometer-sized defects of He^+^-irradiated hydrocarbon films^11^, single-crystalline 6H-SiC^12^ and polycrystalline W materials^13^. For the CAFM measurement, one laser system was used to keep the constant deflection of the PtIr-coated tip in the contact with the measured specimen. A constant voltage (V_tip_) was applied between the PrIr-coated AFM tip and the specimen. From CAFM measurements, we can simultaneously obtain the surface topography and current emission images of irradiated materials at a typical scan rate of 0.30 Hz. When the conductive tip was scanned across the surface of irradiated materials, the difference in the electron emission intensity across the surface was obtained from the current image. The method can be used to efficiently compare the surface microstructures with the distribution of defects or He bubbles in the near-surface layer. The CAFM method is very sensitive to a change in microstructure of measured specimens, and it does not make any damage to the irradiated materials. Each irradiated W specimen was detected for at least 6 times at different locations, and all the measurements have good repeatability.

Our density functional theory (DFT) calculations were performed using the VASP code and ultrasoft pseudopotentials^14^,^15^. The generalized gradient approximation was used for the exchange-correction energy^16^. The calculations were carried out with a plane-wave energy cutoff of 500 eV in 128-atom supercells with the theoretical lattice constant of 3.176 Å, using a 3 × 3 × 3 *k*-point mesh for integration over Brillouin zone^17^. No symmetry constraints were imposed for the optimization of He arrangement between {101} planes. The geometric relaxation was terminated with a force criteria of 10 meV/Å.

Figure 1 shows the SEM images of polycrystalline W specimens non-irradiated (a) and irradiated at He^+^ fluences of (b) 1.0 × 10^25^ /m^2^, (c) 3.0 × 10^25^ /m^2^, (d) 1.0 × 10^26^ /m^2^, (e) 3.0 × 10^26^ /m^2^, and (f) 1.0 × 10^27^ /m^2^. The insert in Fig. 1(b) shows the magnified SEM images. The non-irradiated W exhibits a smooth surface after mechanical polishing (Fig. 1(a)). It can be clearly observed that the surface microstructure of irradiated polycrystalline W is significantly affected by the He^+^ fluence. Plenty of micropores with diameters of a few nanometers are formed at the surface of W specimens irradiated with a He^+^ fluence of 1.0 × 10^25^ /m^2^ (Fig. 1(b)). These micropores are almost uniformly distributed over the W specimen. The W specimen irradiated with a He^+^ fluence of 3.0 × 10^25^ /m^2^ exhibits the wave-like surface microstructure (Fig. 1(c)), and the wavy-like structure appears to be thin strips of material orientated in a certain direction. This indicates that serious surface etching is formed due to He^+^ irradiation. Figure 1(d) shows that the cross-linking wave-like microstructures are formed after W specimen is irradiated with a He^+^ fluence of 1.0 × 10^26^ /m^2^. This indicates that surface etching is enhanced when He^+^ fluence is increased. Our EBSD analysis shows that the wave-like structure orientated in a certain direction has consistent crystallographic orientation, and they are formed at each crystal grain of polycrystalline W (EBSD images of the W specimen irradiated with a He^+^ fluence of 1.0 × 10^26^ /m^2^ were shown in Figure S1 in supporting information). The characteristics of wave-like structures such as roughness appear to strongly depend on the crystallographic orientation of W.

Figure 1(e) shows that the nano-fuzzes are formed along the wave-like microstructures after the W specimen is irradiated with a He^+^ fluence of 3.0 × 10^26^ /m^2^. The He^+^ irradiation at the fluence of 1.0 × 10^27^ /m^2^ results in a high density of nano-fuzzes and nanometer-sized trenches, as shown in Fig. 1(f). These nano-fuzzes are almost uniformly distributed over the W surface while nanometer-sized trenches are orientated in one certain direction. This indicates that the surface swelling of W materials orientated in one certain direction can result in the growth of nano-fuzzes, accompanied by the formation of nanometer-sized trenches.

Figure 2 depicts the surface topography (left) and simultaneously measured current images (right) of W specimens non-irradiated (a) and irradiated with He^+^ fluences of (b) 1.0 × 10^25^ /m^2^, (c) 3.0 × 10^25^ /m^2^, (d) 1.0 × 10^26^ /m^2^, and (e) 3.0 × 10^26^ /m^2^. The inserts in Fig. 2(b,c) show the magnified current images. CAFM measurements have been performed at V_tip_ = −3.6 mV. Marking the locations in Fig. 2(c,d) with dotted lines is to demonstrate the correlation between the surface topography and simultaneously measured current images. The surface of non-irradiated polycrystalline W is relatively smooth (Fig. 1(a)), which is in good consistence with the SEM measurement. The current image in Fig. 1(a) shows that low-density defects exist in the non-irradiated polycrystalline W, which influence the local electron emission of W. The surface topography in Fig. 2(b) shows that nanometer-sized protrusions are formed due to the He^+^ irradiation at the fluence of 1.0 × 10^25^ /m^2^. The SEM image in Fig. 1(b) shows plenty of micropores for the W specimen irradiated with a He^+^ fluence of 1.0 × 10^25^ /m^2^. The characteristic shape and distribution of nanometer-sized defects are observed from the current image (Fig. 2(b)). This indicates that nanometer-sized defects can be formed in the implanted layer due to He^+^ irradiation, resulting in micropores of W materials^13^. Nanometer-sized defects containing He atoms can affect the local electron emission through irradiated W^13^. Impurity atoms, or dislocations can be formed in near-surface layers during the mechanical polishing, and they can act as trapping centers for He atoms^6^,^9^. The formation of nanometer-sized defects can be attributed to the diffusion and coalescence of He atoms into the dislocations or defects. He ions have been found to cause significant changes in W surface microstructures despite the fact that the sputter threshold of W is above the He ion energy of 70 eV^4^. Such surface changes have been proposed to result from the continuous He trapping into nanometer-sized defect sites, which are uniformly distributed in the near-surface layer of W materials^4^. When He atoms are injected into the W material, these nanometer-sized defects can grow as bubbles by pushing out the host atoms from their lattice sites^18^.

After W specimens are irradiated with a He^+^ fluence of 3.0 × 10^25^ /m^2^ and 1.0 × 10^26^ /m^2^, the wave-like microstructures are formed at the W surface, as shown in Fig. 2(c,d). The current images in Fig. 2(c,d) show that one face of wave-like structures is relatively conductive and is full of nanometer-sized defects. The other face of wave-like structures contains a much lower density of defects. This finding indicates that He^+^ ions prefer to penetrate into one certain face of W crystal grains, resulting in the growth of nanometer-sized defects. An increase in the internal pressure of He-containing defects can lead to the formation of dislocations by punching-out process, which affects the distribution of W atoms in crystal grains and local electron emission of irradiated W. The generation of conductive defects indicates that the local W density of nanometer-sized defects is increased during the punching-out process. The formation of wave-like microstructures can be attributed to the continuous etching of crystal grains along one certain face with a high density of nanometer-sized defects. After the W specimen is irradiated with a He^+^ fluence of 3.0 × 10^26^ /m^2^, the wave-like microstructures become denser, and the density of nanometer-sized defects on both sides of wave-like microstructures is obviously increased, as shown in Fig. 2(e). This indicates that an increase in He^+^ fluence results in the strong penetration of He atoms into the W surface layer and strong sputtering of W crystal grains along one certain crystal face. Our measurements show that the CAFM images of the W specimen irradiated with a He^+^ fluence of 1.0 × 10^27^ /m^2^ are unclear because of a high density of nano-fuzzes formed at the W surface.

Our EBSD measurements show that wave-like microstructures are formed at each W crystal grain, depending on the He^+^ fluence. The low-energy (70 eV) He^+^ ions can penetrate deeply into the W surface layer since their diffusion is greatly improved at a relatively high W surface temperature^19^. Thus, the stability of He arrangements in W crystals has to be considered. The generation of wave-like microstructures and nano-fuzzes can be affected by the spatial distribution of W atoms in each crystal grain. In order to analyze the influence of W crystal orientations on the arrangement of He atoms in W crystal grains, DFT calculations have been performed, and the stabilities of interstitial He clusters formed between {100}, {101} and {111} planes are compared. The reliability of the present calculations has been validated in the previous study, indicating that He atom prefers to occupy tetrahedral interstitial (*tet*) sites^20^. When the first He atom occupies a *tet* site, the surrounding *tet* sites have been tested for the second He atom to find the stable configuration of 2-He interstitial cluster. The binding energy of the stable 2-He interstitial cluster is calculated to be 1.07 eV, which agrees well with the result of 1.03 eV obtained by Becquart *et al.*^21^. The possible *tet* sites in vicinity of 2-He cluster are examined to determine the most stable site for the third He atom. In a similar way, more He atoms are brought in pure W to form a larger interstitial He cluster. The interstitial He clusters containing 9 He atoms are plotted in Fig. 3. Our calculations show that He atoms are much more preferable to aggregate between {101} planes compared with other planes such as {100} and {111}. From the microstructure, the He atoms positioned between {100} and {111} planes will diffuse spontaneously to the inter-plane of {101} planes. The total binding energies of interstitial He clusters between {100}, {101} and {111} planes are displayed in Fig. 4. Here, the He atoms between {100} and {111} are fixed at *tet* site, then the stable configurations were found. The energetic calculations demonstrate that He atoms are much more favorable to cluster in a close-packed arrangement between {101} planes.

The previous TEM analysis by Johnson *et al.*^22^,^23^ has shown that the He^+^ implantation into gold (Au) and molybdenum (Mo) can result in the formation of an ordered array – a superlattice of small (<60 nm in diameter) He bubbles. In Au, the superlattice of He bubbles has the same symmetry as the atomic lattice of Au. In Mo, considerable bubble ordering is evident with bubble rows parallel to {101} directions^23^. After the penetration of incident He atoms into W, they may diffuse along the near-surface layer of W crystal grains, particularly at an elevated temperature. In a fully three-dimensional W lattice, the {101} planes have higher areal density and larger spacing than crystal planes in other directions. Our DFT calculation predicts that He atoms prefer to form mono-layer He clusters between {101} planes in single crystal W. The formation of He clusters or defects can be strongly dependent on He^+^ fluence and W surface temperature^3^,^4^,^5^,^6^. Due to favorable energetics, a number of He clusters can be formed between {101} planes with increasing He accumulation at elevated temperature (1400 K), resulting in He-enriched strips, as shown in Fig 5(a). Since He is a closed-shell atom, the binding energy between {101} planes can be greatly reduced due to the existence of He clusters between the planes. With increasing He content, He clusters can aggregate and grow by pushing out the host atoms from their lattice sites^18^. The trapped He clusters result in progressively larger lattice distortions. The penetration of He atoms into the {101} planes reduces the binding forces along those planes, which gives rise to an enhanced sputtering yield along those planes. The strong sputtering of the {101} dense-packed planes can occur, resulting in the release of He atoms, as shown in Fig 5(b). Thus, the wave-like or terraced surface microstructures can be formed due to the sputtering.

The {101} dense-packed planes in the near-surface layer of crystal grains can be exposed during the sputtering, as shown in Fig 6(a). A number of mono-layer He clusters are formed between these dense-packed planes. These He-enriched {101} strips at each crystal grain are almost parallel due to the sputtering along {101} faces. He diffusion in W is strongly dependent on the W surface temperature^19^. Therefore, He atoms can penetrate deeper into the He-enriched {101} planes at a temperature of 1400 K. With increasing He accumulation, He-enriched {101} strips form through the continuous coalescence of He atoms along the {101} planes. The He-enriched surface layer becomes thicker, and the internal pressure of He clusters is increased. It has been proposed that the penetration of He atoms into the near-surface may result in the surface swelling of W because of the high pressure of He clusters or bubbles^13^ An increase in the internal pressure of He-enriched {101} strips results in mutation of crystal lattice, leading to surface swelling, exfoliation, and cleavage of He-enriched {101} strips. Figure 6(b) shows that nano-fuzzes are formed during the surface swelling and cleavage, accompanied by the generation of the nanometer-sized trenches. Thus, nano-fuzzes grow around nanometer-sized trenches, which can be observed from the SEM measurements. With further increasing the He fluence, the density of He-enriched {101} strips is greatly improved, resulting in a high density of nano-fuzzes and nanostructured trenches uniformly distributed over the surface of each crystal grain (Fig. 6(c)). This analysis indicates that the low W resistance to the formation of nano-fuzzes can be attributed to the diffusion and coalescence of He atoms along dense-packed {101} planes at an elevated temperature. Thus, the design of W materials with ultrafine grain structure or defects might be necessary to efficiently avoid the diffusion and coalescence of He atoms along the crystal planes and improve the W resistance to He^+^ irradiation in the fusion reactor.

In summary, He^+^ irradiation of crystalline W at 1400 K results in wave-like surface microstructures or nano-fuzzes, depending on He^+^ fluence. CAFM measurements indicate the existence of the He-enriched strips formed due to He^+^ irradiation. The formation of wave-like microstructures and nano-fuzzes can be attributed to the sputtering and surface swelling of He-enriched trips, respectively. DFT calculations predict that the formation of He-enriched trips is due to He clustering between close-packed {101} planes. To improve the W resistance to He^+^ irradiation in the fusion reactor, W materials with ultrafine grain structure or defects should be designed to effectively avoid the diffusion and coalescence of He atoms along the crystal planes and the growth of nano-fuzzes.

## Methods

The large-power RF plasma system newly built in our lab was utilized to irradiate these W specimens. In this system, large-power (P_max_ = 10 kW) RF plasmas were generated inside the quartz tube (10 cm in diameter, 18 cm in length) by inductive coupling of 2 MHz RF to the water-cooled RF coil, as shown in Figure S2 in supporting information. The insulation of the water-cooled RF coil was provided by polytetrafluoroethylene (PTFE) tube. The quartz tube was subjected to very high thermal loads during the operation of large-power RF plasmas. A water-cooled stainless-steel Faraday screen was utilized to protect the quartz tube from the heat load of the plasma. The permanent magnets installed on the bottom of the quartz tube created a magnetic field in the order of 80 mT and enhance the plasma confinement. Between the plasma source and the main vacuum chamber, one plasma grid with 8 mm holes was installed to confine the high-density plasmas to the quartz tube. The molecular pump was utilized to pump the main vacuum chamber, and the base pressure was 3 × 10^−4^ Pa.

## Additional Information

**How to cite this article**: Yang, Q. *et al.* Nanostructured fuzz growth on tungsten under low-energy and high-flux He irradiation. *Sci. Rep.* **5**, 10959; doi: 10.1038/srep10959 (2015).

## Supplementary Material

Supplementary Information

## Figures and Tables

**Figure 1:**
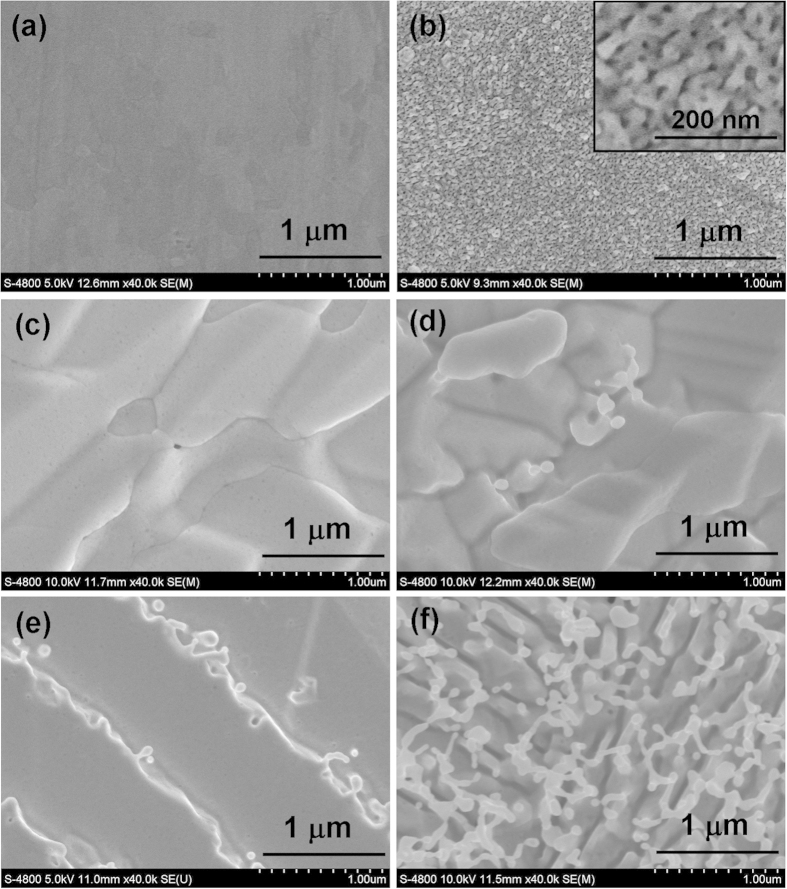
SEM images of polycrystalline W specimens non-irradiated (**a**), and irradiated with He^+^ fluences of (**b**) 1.0 × 10^25^ /m^2^, (**c**) 3.0 × 10^25^ /m^2^, (**d**) 1.0 × 10^26^ /m^2^, (**e**) 3.0 × 10^26^ /m^2^, and (**f**) 1.0 × 10^27^ /m^2^. The insert in Fig. 2(b) shows the magnified SEM images.

**Figure 2:**
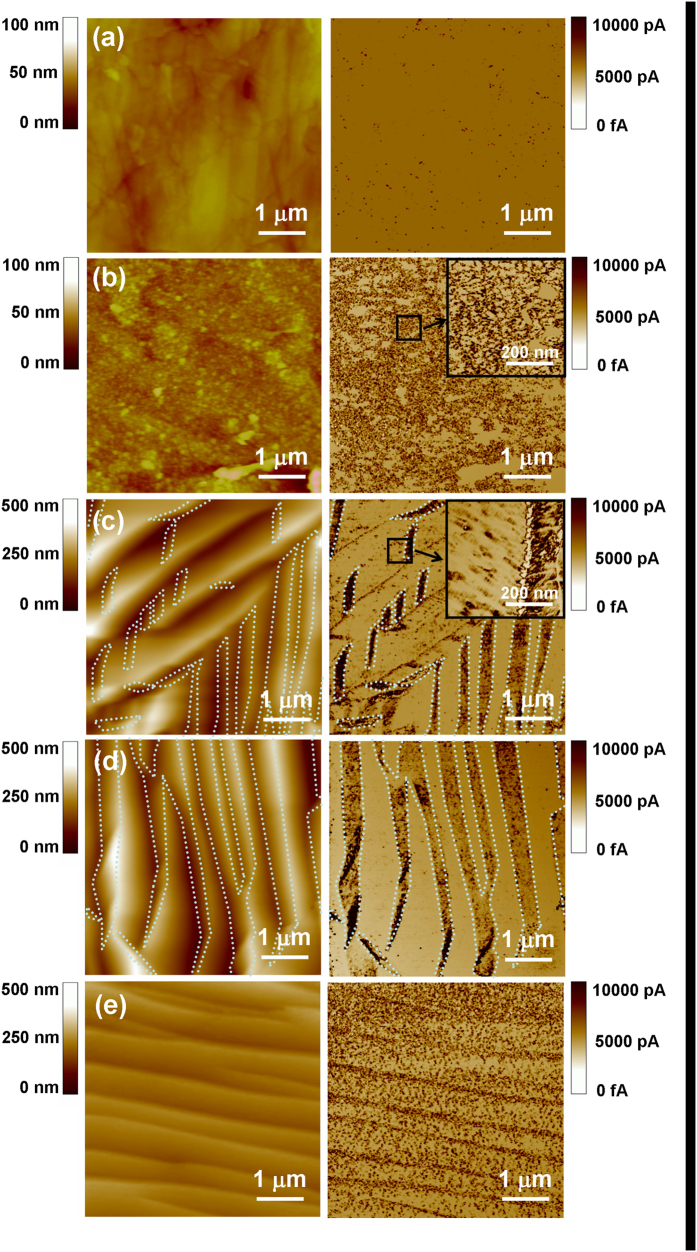
The surface topography (left) and simultaneously measured current images (right) of W specimens non-irradiated (**a**), and irradiated with He^+^ fluences of (**b**) 1.0 × 10^25^ /m^2^, (**c**) 3.0 × 10^25^ /m^2^, (**d**) 1.0 × 10^26^ /m^2^, and (**e**) 3.0 × 10^26^ /m^2^. The inserts in Fig. 2(a,b) shows the magnified current images. CAFM measurements have been performed at V_tip_= −3.6 mV.

**Figure 3:**
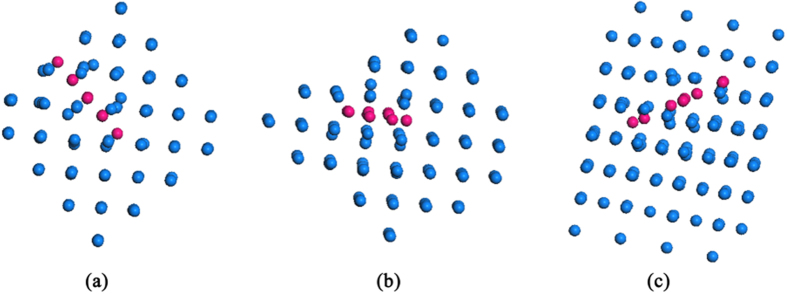
The interstitial He clusters containing 9 He atoms formed between (**a**) {100} planes, (**b**) {101} planes, and (**c**) {111} planes. The blue and pink balls represent tungsten and He atoms, respectively.

**Figure 4:**
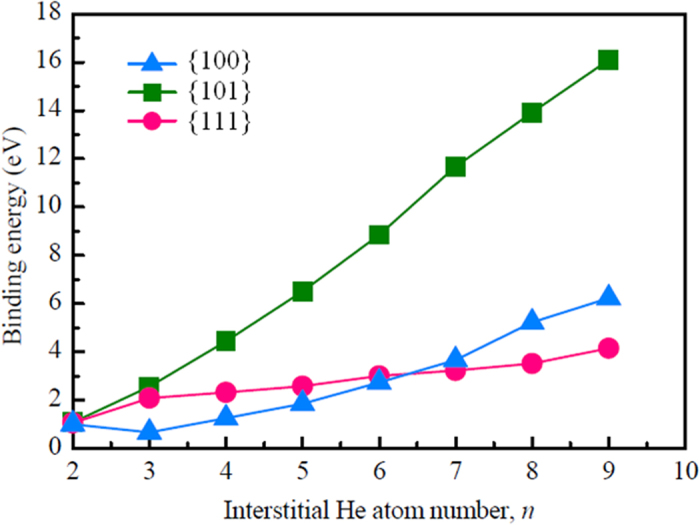
The binding energies of interstitial He clusters between {100} planes, {101} planes, or {111} planes as a function of He atom number in bcc tungsten.

**Figure 5:**
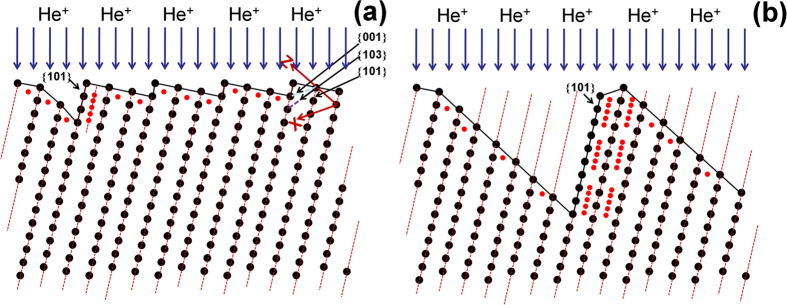
The formation process of wave-like microstructures at the W crystal grain due to the surface sputtering of He-enriched {101} dense-packed planes. When the W crystal grain is exposed to low-energy He ions, they penetrate into the near-surface and diffuse along the surface layer at an elevated temperature. (**a**): He atoms prefer to form mono-layer He clusters between {101} planes in single crystal W and form He-enriched strips containing a high density of He clusters. Since He is a closed-shell atom, the binding energy between {101} planes can be greatly reduced due to the existence of He clusters between the planes. (**b**): The strong sputtering of the {101} dense-packed planes can occur due to the existence of He clusters, resulting in the release of He atoms. The wave-like surface microstructures can be formed at the W crystal grain.

**Figure 6:**
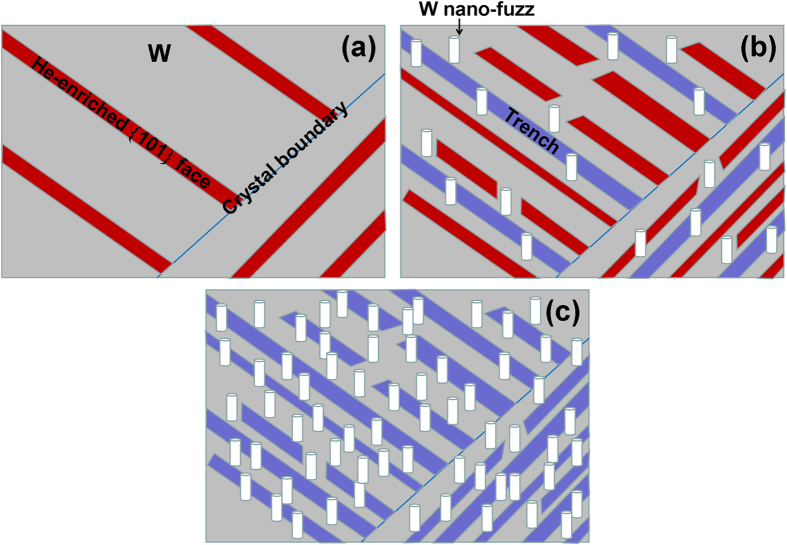
Nano-fuzz growth of polycrystalline W exposed to low-energy He ions. (**a**): The {101} dense-packed planes containing a high density of mono-layer He clusters can be formed at the near surface of crystal grains due to He^+^ bombardments. He-enriched {101} strips form through the continuous coalescence of He atoms between the {101} planes. (**b**): An increase in the pressure of He clusters result in mutation of crystal lattice, leading to surface swelling of the He-enriched {101} strips. Nano-fuzzes are formed during the swelling, accompanied by the generation of nanostructured trenches. (**c**): With increasing He^+^ fluence, more He-enriched {101} strips are formed, resulting in a high density of nano-fuzzes and nanostructured trenches uniformly distributed over the surface of each crystal grain.
