# Supplementary material for: Nanostructured fuzz growth on tungsten under low-energy and high-flux He irradiation
Source: Sci Rep. 2015 Jun 16;5:10959. doi: 10.1038/srep10959 (PMC4468520; doi:10.1038/srep10959)
Supplement: Supplementary Information [file srep10959-s1.doc]

Nano-fuzz growth of crystalline tungsten exposed to low-energy and high-flux He ions

Qi Yang,^1^ Yu-Wei You,^2^ Lu Liu,^1^ Hongyu Fan,^1^ Weiyuan Ni,^1^ Dongping Liu,1,3,[[1]](#footnote-2)* C. S. Liu,^2,*^ Günther Benstetter^4^ & Younian Wang^5^

*^1^School of Physics and Materials Engineering, Dalian Nationalities University, Dalian 116600, People’s Republic of China*

*^2^Key Laboratory of Materials Physics, Institute of Solid State Physics, Chinese Academy of Sciences, P. O. Box 1129, Hefei 230031, P. R. China*

*^3^Fujian Key Laboratory for Plasma and Magnetic Resonance, Department of Electronic Science, Aeronautics, School of Physics and Mechanical & Electrical Engineering, Xiamen University, Xiamen, 361005, People’s Republic of China*

*^4^Faculty of Electrical Engineering and Media Technology, University of Applied Sciences Deggendorf, Deggendorf 94469, Germany*

*^5^School of Physics and Optoelectronic Technology, Dalian University of Technology, Dalian 116024, People’s Republic of China*


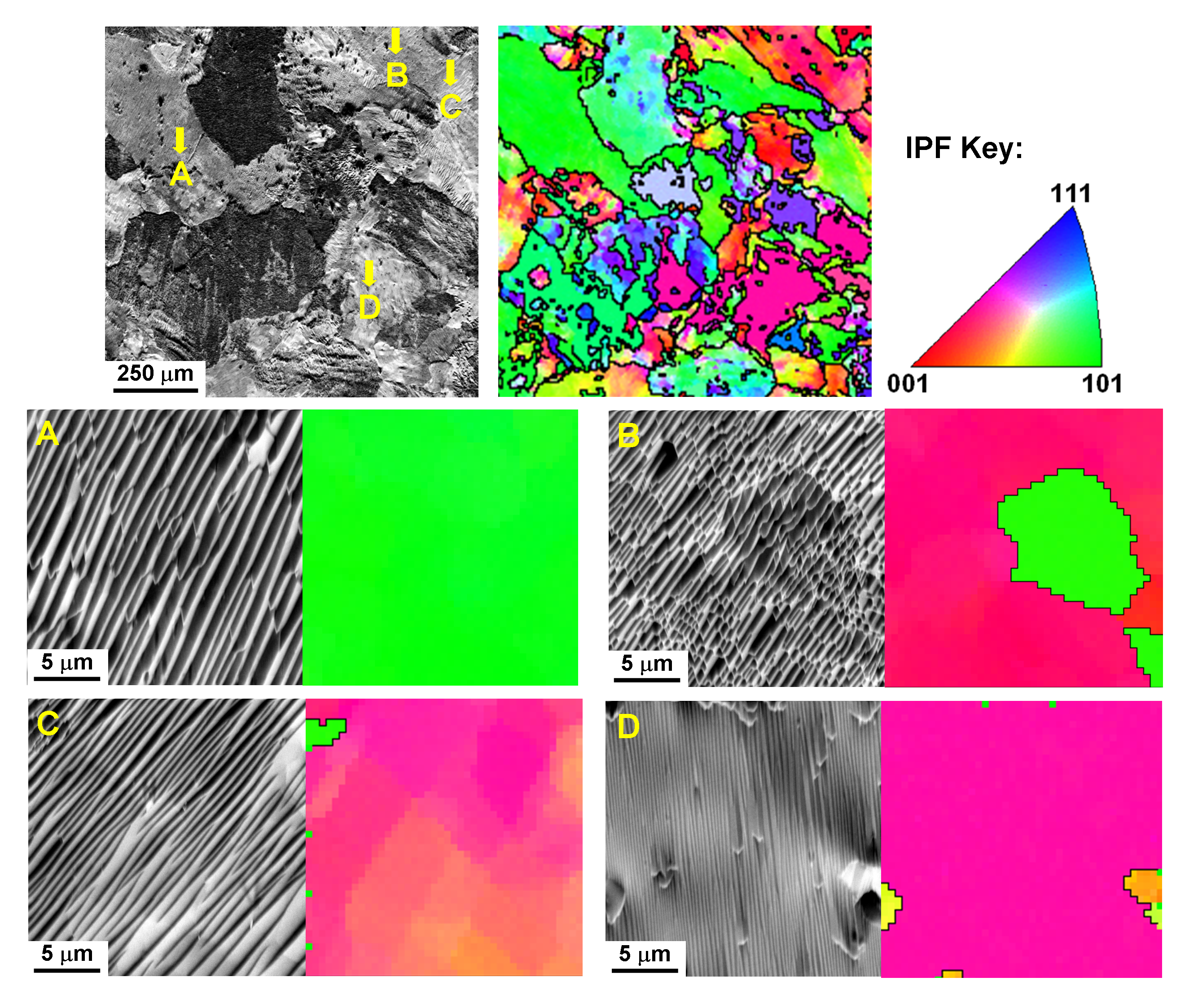


Figure S1 SEM images of polycrystalline W irradiated with 70 eV-He^+^ to a fluence of 1.0×10^26^/m^2^ at 1400 K, and corresponding EBSD mappings.


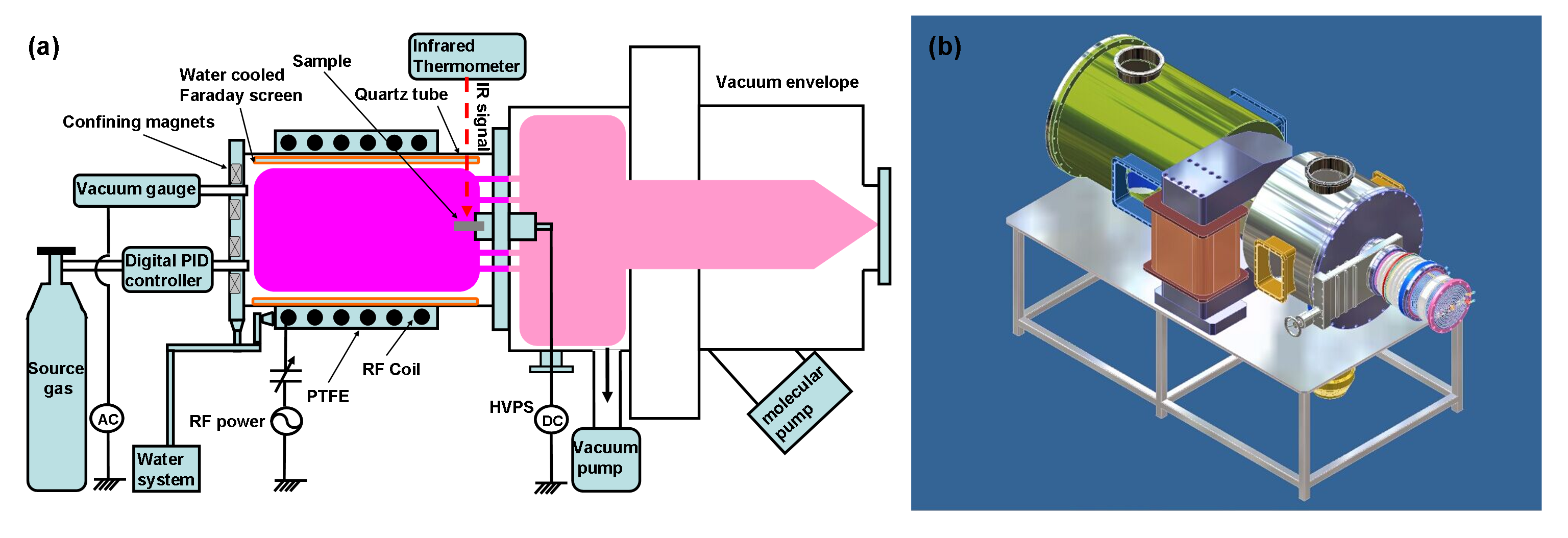
Figure S2 Schematic view (a) and isometric view (b) of large-power RF plasma irradiation system.

1. ^^ Corresponding authors. Tel.:+86-411-87508902; Fax: +86-411-87656331.

   Email address: [dongping.liu@dlnu.edu.cn](mailto:dongping.liu@dlnu.edu.cn) (D.P. Liu) ; [csliu@issp.ac.cn](mailto:csliu@issp.ac.cn)(C.S. Liu). [↑](#footnote-ref-2)
